# Heightened Salience of Anger and Aggression in Female Adolescents With Borderline Personality Disorder—A Script-Based fMRI Study

**DOI:** 10.3389/fnbeh.2018.00057

**Published:** 2018-03-26

**Authors:** Marlene Krauch, Kai Ueltzhöffer, Romuald Brunner, Michael Kaess, Saskia Hensel, Sabine C. Herpertz, Katja Bertsch

**Affiliations:** ^1^Department of General Psychiatry, Center for Psychosocial Medicine, University of Heidelberg, Heidelberg, Germany; ^2^Department of Psychology, Goethe University Frankfurt, Frankfurt am Main, Germany; ^3^Bernstein Center for Computational Neuroscience, University of Heidelberg, Mannheim, Germany; ^4^Department of Child and Adolescent Psychiatry, Center for Psychosocial Medicine, University of Heidelberg, Heidelberg, Germany; ^5^University Hospital of Child and Adolescent Psychiatry and Psychotherapy, University of Bern, Bern, Switzerland; ^6^Department of Psychosomatic Medicine, Central Institute of Mental Health, Mannheim, Germany

**Keywords:** anger, aggression, emotion regulation, borderline personality disorder, adolescence, social threat sensitivity, functional magnetic resonance imaging, group x age interaction

## Abstract

**Background:** Anger and aggression belong to the core symptoms of borderline personality disorder. Although an early and specific treatment of BPD is highly relevant to prevent chronification, still little is known about anger and aggression and their neural underpinnings in adolescents with BPD.

**Method:** Twenty female adolescents with BPD (age 15–17 years) and 20 female healthy adolescents (age 15–17 years) took part in this functional magnetic resonance imaging (fMRI) study. A script-driven imagery paradigm was used to induce rejection-based feelings of anger, which was followed by descriptions of self-directed and other-directed aggressive reactions. To investigate the specificity of the neural activation patterns for adolescent patients, results were compared with data from 34 female adults with BPD (age 18–50 years) and 32 female healthy adults (age 18–50 years).

**Results:** Adolescents with BPD showed increased activations in the left posterior insula and left dorsal striatum as well as in the left inferior frontal cortex and parts of the mentalizing network during the rejection-based anger induction and the imagination of aggressive reactions compared to healthy adolescents. For the other-directed aggression phase, a significant diagnosis by age interaction confirmed that these results were specific for adolescents.

**Discussion:** The results of this very first fMRI study on anger and aggression in adolescents with BPD suggest an enhanced emotional reactivity to and higher effort in controlling anger and aggression evoked by social rejection at an early developmental stage of the disorder. Since emotion dysregulation is a known mediator for aggression in BPD, the results point to the need of appropriate early interventions for adolescents with BPD.

## Introduction

Borderline Personality Disorder (BPD) is a life-span mental disorder, which causes a high burden for the affected individuals and their social environment. Patients with BPD are characterized by instability in affect and interpersonal relationships, identity disturbance, and heightened impulsivity. Furthermore, deficits in emotion regulation as well as intense feelings of anger and difficulties in anger control belong to the core symptoms of BPD [American Psychiatric Association (APA), 2013]. In an effort to cope with strong negative feelings, such as anger, many BPD patients show self-destructive behaviors such as self-injury that commonly begins already in early adolescence (Zanarini et al., 2008). In addition, aggressive outbursts against significant others are frequent reactions in patients with BPD. These often result from intense feelings of anger provoked by potential signals of interpersonal threat, such as interpersonal provocation, rejection, or exclusion and may therefore be described as predominantly reactive in nature (Newhill et al., 2009, 2012; for review, also see Mancke et al., 2015; Zanarini et al., 2017).

Previous cross-sectional and longitudinal studies have revealed emotion dysregulation and high levels of trait anger as important mediators of increased reactive aggression in BPD (Newhill et al., 2012; Scott et al., 2014; Mancke et al., 2017), thus supporting the theory of aggressive behavior in BPD being a dysfunctional effort to control intense feelings of anger. Typical situational triggers for feelings of anger are interpersonal situations where the patients feel rejected or excluded by others. Consistently it is assumed that patients with BPD are hypersensitive for interpersonal rejection and threats (Barnow et al., 2009; Bertsch et al., 2013, 2017; Veague and Hooley, 2014) and it has been described that this heightened interpersonal threat sensitivity is one of the core mechanisms for reactive aggression in BPD (Mancke et al., 2015). The deficit in coping with feelings of anger is supposed to be due to the patients' more general deficit in regulating intense negative emotions. Several studies have investigated the neural mechanisms that underlie these emotion regulation difficulties in adult patients with BPD and revealed structural as well as functional frontolimbic abnormalities. A meta-analysis of Schulze et al. (2016) confirmed enhanced activation in the left amygdala and the left hippocampus to emotionally negative stimuli in medication-free adult patients with BPD compared to healthy controls. Furthermore, enhanced activation in the left posterior insula could be found in adult patients with BPD (Schulze et al., 2016). While these findings on limbic hyperactivation reflect a heightened emotional responding to negative emotional stimuli, further findings on a hypoactivation in the anterior cingulate cortex (ACC) and prefrontal regions, such as the orbitofrontal cortex (OFC) (Silbersweig et al., 2007; Koenigsberg et al., 2009; Krause-Utz et al., 2014) support the assumption of deficits in regulating limbic activation. Using a script-driven imagery paradigm to study the neural correlates of imagined physical aggressive reactions to rejection-related anger, we recently found elevated right lateral orbitofrontal and right dorsolateral PFC activations in adult male BPD patients compared to both adult female BPD patients and healthy male controls (Herpertz et al., 2017). While female groups did not differ in neural responses to anger induction in this study, enhanced activations in the dorsal anterior cingulate cortex (dACC), medial prefrontal cortex (mPFC), precuneus, and insula were found in female adult BPD patients compared to healthy controls when feelings of social exclusion were induced with a cyberball paradigm (Domsalla et al., 2013).

Although empirical research has confirmed the reliability and validity of BPD among adolescents (Kaess et al., 2014; Winsper et al., 2016), until now only few studies on emotion dysregulation have focused on BPD in childhood and adolescence. However, this group is of particular interest since the investigation of deficits in emotion regulation in adolescents with BPD could give essential insights in the etiology and the course of the disorder and might hence minimize confounding influences in adult samples, such as a long history of illness and comorbidities as well as treatment exposure. It could therefore not only contribute to a better understanding of BPD in general but also to an improvement of early interventions that may attenuate the full manifestation of the disorder.

Interestingly, Lawrence et al. (2011) found that being excluded triggered the same negative emotions in adolescents and young adults with BPD (15–24 years) as in healthy controls, but that the subjective intensity of these negative emotions (amongst others anger) was elevated in the BPD group. Hence, although triggering negative emotions across groups, already in adolescence experiences of social exclusion are associated with stronger arousal and higher emotional intensity in adolescents with BPD. Besides more intense negative emotional reactions to experiences of social exclusion, deficits in the regulation of emotions have been found in adolescents with BPD compared to healthy controls but also adolescents with other psychiatric disorders, using psychometric (Ibraheim et al., 2017) as well as ambulatory assessment methods (Santangelo et al., 2017). On a neural level, the few studies that investigated structural differences in adolescents with BPD point to volume reductions in the OFC (Chanen et al., 2008; Brunner et al., 2010) and the ventral ACC (Whittle et al., 2009; Goodman et al., 2011), but not in the amygdala or hippocampus as reported for adult BPD patients (Schulze et al., 2016). Interestingly, structural brain differences in partly overlapping brain regions could also be revealed at a very early stage of development in other psychiatric disorders related to emotion dysregulation, such as unipolar and bipolar depressive disorders (Serafini et al., 2014). Functional brain alterations in adolescents with BPD have so far only been addressed in one pilot study. Comparing neural responses to emotionally negative pictures in six adolescent patients with BPD and six healthy controls, this study revealed increased activations in the amygdala, hippocampus, superior frontal gyrus, and precentral gyrus in adolescents with BPD (LeBoeuf et al., 2016).

Taken together, previous fMRI studies on emotion dysregulation as well as anger and aggression in BPD have by the majority focused on adult samples and only little is known about the neural correlates of these aspects of the symptomatology in the early stage of the disorder. Considering the formerly reported structural alterations as well as deficient self-reported emotion regulation in adolescents with BPD, we also assume functional abnormalities in brain circuits involved in the regulation of emotions, such as anger, in this early stage of BPD. In improving our understanding of the neural correlates associated with the symptomatology, we hope to contribute to an early implementation of appropriate therapeutic interventions for adolescents with BPD.

The aim of the present study was to investigate neural correlates of rejection-related feelings of anger and of subsequent other-directed or self-directed aggressive reactions in female adolescent BPD patients in order to contribute to a better understanding of the etiology of disturbed emotion regulation in BPD. Given the fact that most of the psychiatric in- and out-patients with BPD is female and most of previous research has focused on female adult and adolescent BPD patients, we decided to focus on an all-female sample in this first fMRI study on anger and aggression in adolescents with BPD knowing the importance of investigating sex-dependent effects in further studies.

We used a script-driven imagery paradigm with scripts describing a social rejection/exclusion situation and the elicited intense feelings of anger followed by descriptions of self-directed aggressive reactions or aggressive reactions against the rejecting person. Based on previous findings in adolescents with BPD and similar to adult BPD patients, we expected a heightened emotional responding in adolescents with BPD compared to an age-matched healthy control group. We hypothesized that the latter would be reflected in a hyperactivation in the amygdala and the insula in adolescents with BPD compared to adolescent healthy controls when listening to the scripts describing anger inducing rejection situations as well as aggressive behavior. Furthermore, we hypothesized to find reduced activations in prefrontal regions, such as the OFC reflecting deficits in the regulation of highly arousing states of negative emotion in adolescents BPD compared to healthy adolescents. Consistently, we expected adolescents with BPD to score higher on the ratings for feelings of anger after listening to the scripts.

## Materials and methods

### Participants

Twenty female adolescents with BPD (Y-BPD; age 15–17 years) and 20 female adolescent healthy controls (Y-HC; age 15–17 years) took part in the study. The study also comprised 34 female adults with BPD (A-BPD; age 18–50 years) and 32 female adult healthy controls (A-HC; age 18–50 years). The adult sample was almost identical with the female sample reported by Herpertz et al. (2017), therefore, the current analyses and results focus on the adolescent sample and age-related differences.

All adolescent and adult BPD patients currently met at least 5 out of 9 BPD criteria according to the Diagnostic and Statistical Manual of Mental Disorders, 4th edition (DSM-IV, American Psychiatric Association, 2013) and all adolescent and adult healthy controls had never received a psychiatric diagnosis or undergone a psychotherapeutic/psychopharmacological treatment (see Table 1 for details regarding the sample characteristics). For the two adolescent groups, participants were included with at least 15 years and at most 17 years of age, for the two adult groups participants had to be 18–50 years old. General exclusion criteria comprised: Neurological disorders, current alcohol/drug abuse (urine toxicology screening), alcohol/drug abuse in the last 2 months (interview) or severe medical illness. Additional exclusion criteria were lifetime diagnoses of schizophrenia, schizoaffective or bipolar disorder, as well as reported alcohol/drug dependence in the last 12 months. In the adolescent group, *N* = 4 patients took antidepressant medication (*N* = 3 SSRIs, *N* = 1 Agomelatin), while all adult patients had been free from any psychotropic medication-use for at least 2 weeks prior to participation.

**Table 1:** Sample description and self-report data.

	**Y-BPD (*****N*** = **20)**		**Y-HC (*****N*** = **20)**		**A-BPD (*****N*** = **34)**		**A-HC (*****N*** = **32)**		**Y-BPD vs. Y-HC**		**group** × **age**	
	**Mean**	**Std**.	**Mean**	**Std**.	**Mean**	**Std**.	**Mean**	**Std**.	***t***	***p***	***F***	***p***
Age (years)	16.35	0.88	15.85	0.81	25.69	5.08	27.33	6.37	*t*_(38)_ = 1.87	*P* = 0.069	*F*_(1, 101)_ = 1.36	*P* = 0.246
Raven	50.55	4.55	52.89	4.65	53.46	4.29	53.40	4.43	*t*_(37)_ = −1.59	*P* = 0.120	*F*_(1, 100)_ = 1.78	*P* = 0.185
ZAN-BPD total score	17.40	5.86	0.00	0.00	11.76	4.86	0.39	0.80	*t*_(19)_ = 13.28	*P* ≤ 0.001	*F*_(1, 101)_ = 15.64	*P* ≤ 0.001
AQ total score	62.75	16.91	41.32	8.08	61.12	11.92	43.39	7.83	*t*_(33)_ = 4.91	*P* ≤ 0.001	*F*_(1, 96)_ = 0.63	*P* = 0.431
STAXI trait anger	21.19	8.31	14.11	4.47	23.82	5.87	14.40	3.58	*t*_(33)_ = 3.21	*p* = 0.003	*F*_(1, 96)_ = 1.02	*P* = 0.315
DERS total score	109.56	34.11	56.00	11.13	123.55	16.14	63.85	14.14	*t*_(17.69_) = 6.02	*P* ≤ 0.001	*F*_(1, 96)_ = 0.60	*P* = 0.442
BIS total score	75.81	14.32	57.22	8.52	77.17	12.09	60.49	10.46	*t*_(23.84)_ = 4.53	*P* ≤ 0.001	*F*_(1, 95)_ = 0.16	*P* = 0.694
FDS total score	20.85	15.39	3.62	3.32	19.26	12.12	2.93	2.35	*t*_(15.04)_ = 4.26	*P* = 0.001	*F*_(1, 95)_ = 0.05	*P* = 0.824
	**Lifetime**	**Current**	**Lifetime**	**Current**	**Lifetime**	**Current**	**Lifetime**	**Current**				
Affective disorders	12 (60%)	10 (50%)	0	0	31 (91%)	11 (32%)	0	0				
Substance ass. disorders	1 (5%)	0 (0%)	0	0	6 (18%)	0 (0%)	0	0				
Anxiety disorders	4 (20%)	5 (25%)	0	0	20 (59%)	15 (44%)	0	0				
PTSD	1 (5%)	1 (5%)	0	0	9 (27%)	8 (24%)	0	0				
Somatoform disorders	0 (0%)	0 (0%)	0	0	5 (15%)	5 (15%)	0	0				
Eating disorders	8 (40%)	4 (20%)	0	0	20 (59%)	13 (38%)	0	0				
Antisocial PD	0 (0%)	0 (0%)	0	0	1 (3%)	1 (3%)	0	0				
Avoidant PD	0 (0%)	0 (0%)	0	0	0 (0%)	0 (%)	0	0				

*Y-BPD, female adolescents with BPD; Y-HC, female adolescent healthy controls; A-BPD, female adults with BPD; A-HC, female adult healthy controls. ZAN-BPD, Zanarini Rating Scale for Borderline Personality Disorder; AQ, Buss and Perry Aggression Questionnaire; STAXI trait anger, trait scale of the State-Trait Anger Expression Inventory; DERS, Difficulties in Emotion Regulation Scale; BIS, Barratt Impulsiveness Scale; FDS, Fragebogen für dissoziative Symptome (FDS), which is the German version of the Dissociative Experiences Scale (DERS); PTSD, Posttraumatic Stress Disorder; PD, personality disorder*.

Recruitment was done by the central project of the KFO 256 (Schmahl et al., 2014), a Clinical Research Unit funded by the German Research Foundation (DFG) dedicated to investigating mechanisms of disturbed emotion processing in BPD. The study was approved by the Ethics Committee of the Medical Faculty of the University of Heidelberg. Participants and caregivers (in case of minors) provided written informed consent.

### Measures

All patients and healthy controls took part in an extensive on-site diagnostic interview to assess BPD and other current and lifetime psychiatric disorders. Interviews consisted of the Structured Clinical Interview for DSM-IV disorders (SCID-I; Wittchen et al., 1997) and the International Personality Disorder Examination (IPDE; Loranger, 1999) for assessing the diagnosis of BPD and axis I and II comorbidities. Interviews were performed by experienced diagnosticians who held at least a Master's degree in Psychology or M.D. and underwent standardized training resulting in high inter-rater reliabilities (ICC ≥ 0.91 for both, the number of BPD criteria and the dimensional score assessed by the ZAN-BPD scale). BPD symptom severity was assessed with the Zanarini Rating Scale (ZAN-BPD; Zanarini et al., 2003). Additionally the following trait measurements were assessed: impulsivity with the Barratt Impulsiveness Scale (BIS; Patton and Stanford, 1995), dissociation with the “Fragebogen zu Dissoziativen Symptomen” (FDS; Freyberger et al., 1999; German version of the Dissociative Experience Scale by Bernstein and Putnam), and emotion dysregulation with the Difficulties in Emotion Regulation Scale (DERS; Gratz and Roemer, 2004), as well as intelligence based on Raven's progressive matrices (Raven et al., 2003). Moreover, trait anger was assessed with the State-Trait Anger Expression Inventory (STAXI; Spielberger, 1991), a factor derived instrument that comprises 44 items measuring the experience as well as the expression of anger. Discriminant and convergent validity have been supported (Deffenbacher et al., 1996). Trait aggressiveness was measured with the Aggression Questionnaire (AQ; Buss and Warren, 2000), which comprises the four scales Anger, Hostility, Verbal Aggression and Physical Aggression. It is a well-established instrument for the assessment of aggression, the four scales have been reported to have moderate to high internal consistencies and to be stable over 7 months of testing (Harris, 1997).

### Script-driven imagery task

We used a script-driven imagery task with participants listening to eight standardized scripts, each consisting of four separate phases: baseline, anger induction, other-directed/self-directed aggression, relaxation. The anger induction phase was based on narratives of interpersonal rejection, the other-directed aggression phase comprised narratives of directing physical aggression toward another person, the self-directed aggression phase narratives of self-harming behavior. Each participant listened to four scripts containing narratives of aggressive behavior against others and four scripts describing aggressive behavior against oneself, with order of presentation of the two script types being pseudo-randomized. Within each script, the duration of each of the four phases was 25 s and the duration of the inter-phase interval was 8 s. The scripts were lively read by professional actors and participants were instructed to imagine the described scenes as vividly as possible in order to provoke intense emotional responses. Each of the eight scripts was followed by self-ratings on 5-point Likert scales asking for feelings of anger after the anger induction phase, feelings of anger after the aggression phase as well as for levels of dissociation, derealization, and vividness of imagination. The self-ratings were followed by a 20 s inter-script interval.

### Data acquisition

Data acquisition was performed in a 3T Tim Trio whole-body scanner (Siemens, Erlangen, Germany) equipped with a 32-channel head coil. Forty transverse slices were acquired in each volume using a T2^*^-sensitive gradient EPI sequence (*TR* = 2.350 s, *TE* = 27 ms, voxel size 2.3 × 2.3 × 2.3 mm). Additionally, isotropic high-resolution (1 × 1 × 1 mm) T1-weighted coronal-oriented structural images were recorded. The course of the experiment as well as acquisition of the data during the experiment was controlled by Presentation 14.2 (Neurobehavioral Systems). Audio texts were presented using an in-ear sound system (Sensimetrics).

### Data analysis

#### Self-report and self-rating data

Self-report and self-rating data were analyzed using SPSS 20.0 using *t*-tests for independent groups (Y-BPD vs. Y-HC) with a two-tailed *p* < 0.05. We additionally performed 2 × 2 analyses of variance (ANOVAs) to analyze specific characteristics of the adolescent BPD sample (group by age interactions). Please note that varying degrees of freedom are due to missing data from one A-HC for ZAN-BPD, from four Y-BPD, one Y-HC, and one A-HC for all other questionnaire data, and from one Y-HC and one A-HC for the self-ratings during the experiment.

#### FMRI data

FMRI data were preprocessed and analyzed in SPM8 under Matlab R2012b. Standard data preprocessing comprised temporal adjustment for differences in slice time acquisition, motion correction, co-registration of EPI images with T1-weighted structural images, segmentation of structural images, normalization into MNI space, and spatial smoothing with an 8-mm full-width-half-maximum (FWHM) kernel. On the first level we set up a general linear model (GLM) for each participant with baseline, anger, other-directed aggression, self-directed aggression, relaxation and rating as regressors as well as 6 motion regressors; we defined the contrasts baseline, anger, other-directed aggression and self-directed aggression for each participant. On the second level, we entered these contrasts into a group (BPD, HC) × age (adolescent, adult) × phase (baseline, anger, other-directed aggression, self-directed aggression) full-factorial model. Since we were primarily interested in neural correlates of anger and aggression in adolescents with BPD and differences between adults with and without BPD are reported elsewhere (Herpertz et al., 2017), the reported analyses focus on differential contrast between Y-BPD and Y-HC (i.e., Y-BPD vs. Y-HC for anger>baseline, other directed aggression > baseline, and self-directed aggression > baseline). The specificity of the reported effects for the adolescent sample was addressed in additional group (BPD vs. HC) by age (adolescent vs. adults) interaction analyses. To protect against false positive activations, we used a double-threshold approach in all of our fMRI analysis, combining a voxel-based threshold (*p* < 0.001, uncorrected) with a minimum cluster size of *k* ≥ 88 (Hayasaka and Nicols, 2004). For this purpose we first used the AFNI program 3dFWHMx to estimate the parameters of an extended, mixed model spatial autocorrelation function (ACF) on the basis of our data. We entered the resulting parameters (a, b, c) 0.313954, 5.59137, 10.2879 into 3dClustSim and obtained a minimum cluster size of 87.7 voxels for our current data, given an uncorrected single voxel threshold of *p* < 0.001 and a corrected cluster based threshold of *p* < 0.05.

## Results

### Self-report data

Adolescents with BPD reported significantly higher levels of BPD symptoms, aggressiveness, trait anger, emotion dysregulation, impulsivity, and dissociation than healthy adolescent controls [all *t*_(33)_ ≥ 3.21, *p* ≤ 0.003]. The group by age interaction was significant for symptom severity [*F*_(1, 101)_ = 15.64; *p* ≤ 0.001; $\eta_{\text{p}}^{2}$ = 0.13; higher symptom severity in Y-BPD vs. A-BPD; *t*_(52)_ = −3.81; *p* ≤ 0.001]. Further details on demographic, diagnostic, and self-report data are provided in Table 1.

### Self-ratings

Adolescents with BPD did not differ significantly from healthy adolescent controls in their subjective anger ratings after the rejection-based anger induction phase or the aggression phase, the vividness of imagination, or subjective derealization [*t*_(39)_ ≤ 1.92, *p* ≥ 0.062], but Y-BPD reported significantly stronger dissociation than Y-HC [*t*_(39)_ ≤ 2.50, *p* ≥ 0.017]. There were no significant group by age interactions except for subjective anger after aggression [*F*_(1, 114)_ = 4.26; *p* = 0.041] with significantly higher anger ratings in healthy adolescents than healthy adults [*t*_(46)_ = −2.07; *p* = 0.045], but no significant difference between adolescents and adults with BPD [*t*_(40.87)_ = 0.11; *p* = 0.913]. Details about self-rating data are provided in Table 2.

**Table 2:** Self-rating data.

	**Y-BPD (*****N*** = **20)**		**Y-HC (*****N*** = **19)**		**A-BPD (*****N*** = **34)**		**A-HC (*****N*** = **31)**		**Y-BPD vs. Y-HC**		**Group x**	**Age**
	**Mean**	**Std**.	**Mean**	**Std**.	**Mean**	**Std**.	**Mean**	**Std**.	***t***	***p***	***F***	***p***
Anger after provocation	3.54	0.87	3.63	0.54	3.66	0.63	3.24	0.98	−0.58	0.564	1.29	0.259
Anger after aggression	3.43	1.04	3.23	0.87	3.64	0.79	2.69	1.04	0.65	0.522	4.26	0.041
Derealisation	2.28	1.02	1.74	0.80	2.26	1.04	1.42	0.81	2.06	0.046	1.37	0.244
Dissociation	2.13	1.09	1.36	0.60	2.06	0.97	1.26	0.71	3.04	0.005	0.10	0.757
Vividness of imagination	2.89	0.99	3.34	1.09	2.86	0.85	2.52	1.34	−1.51	0.139	3.04	0.084

*Y-BPD, female adolescents with BPD; Y-HC, female adolescent healthy controls; A-BPD, female adults with BPD; A-HC, female adult healthy controls*.

### fMRI data

#### Anger-induction phase

**Figure 1:**
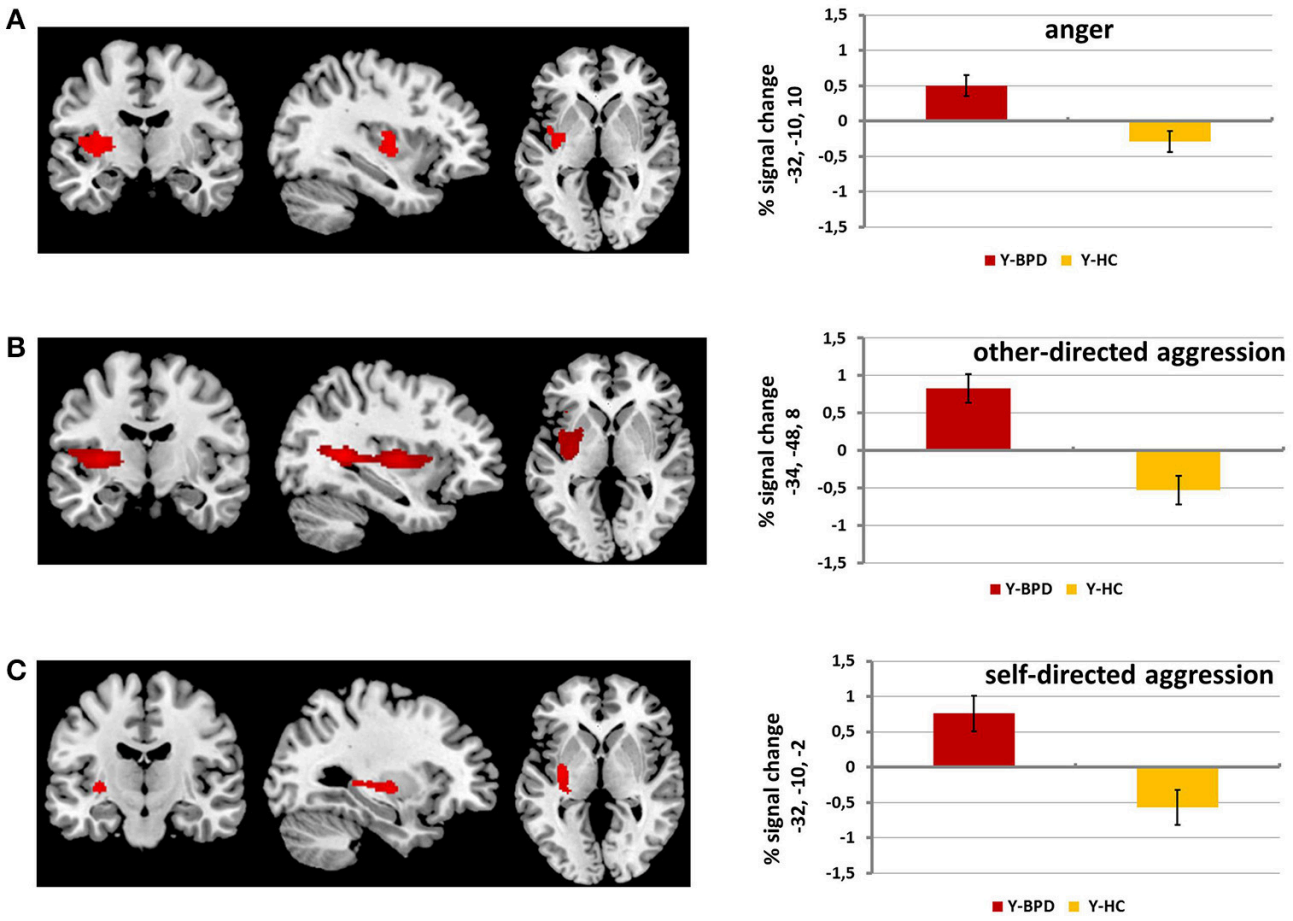
fMRI results for anger, other-directed aggression and self-directed aggression phase. This figure displays whole brain results (*p* < 0.001 and cluster size *k* ≥ 88) for the contrast adolescents with BPD (Y-BPD) vs. healthy adolescents (Y-HC) for anger phase vs. baseline phase **(A)**, other-directed aggression phase vs. baseline phase **(B)** and for self-directed aggression phase vs. baseline phase **(C)**.

Y-BPD showed higher activation in a large cluster comprising parts of the left insula, putamen and claustrum (peak voxel [x, y, z]: −32, −10, 10; *T* = 3.62, *k* = 394, *p* < 0.001) compared to Y-HC. The reverse contrast (Y-HC>Y-BPD) did not result in any significant effects (see Table 3 and Figure 1A). The group by age interactions revealed a significant cluster in the left postcentral gyrus and the left precuneus ((Y-BPD>Y-HC)>(A-BPD>A-HC) peak voxel [x, y, z]: −30, −36, 68; *T* = 4.20, *k* = 102, *p* < 0.001).

**Table 3:** Full-Factorial Analysis, whole brain results during anger induction phase, *p* < 0.001 and cluster size *k* ≥ 88.

		***k***	***p***	***T***	***Z***	**MNI (peak voxel)**		
						***x***	***y***	***z***
Y-BPD > Y-HC	Left insula, left putamen, left rolandic operculum, claustrum	394	<0.001	3.62	3.59	−32	−10	10
Y-HC > Y-BPD (Y-BPD>Y-HC) > (A-BPD>A-HC)	No significant results left postcentral gyrus, left precuneus	102	<0.001	4.20	4.15	−30	−36	68
(Y-HC>Y-BPD) > (A-HC>A-BPD)	No significant results							

*Y-BPD, female adolescents with BPD; Y-HC, female adolescent healthy controls; A-BPD, female adults with BPD; A-HC, female adult healthy controls; k, cluster size; MNI, location of the peak voxel according to the Montreal Neurological Institute brain atlas*.

#### Other-directed aggression phase

Y-BPD showed higher activation in a large cluster including parts of the left insula, putamen, opercular part of the inferior frontal gyrus, middle and superior temporal gyri, pallidum, precuneus, thalamus, and hippocampus (peak voxel [x, y, z]: −34, −48, 8; *T* = 5.03, *k* = 1963, *p* < 0.001; see Table 4 and Figure 1B) compared to Y-HC. The group by age interaction revealed a similar cluster ((Y-BPD>Y-HC)>(A-BPD>A-HC) peak voxel [x, y, z]: −30, −12, 8; *T* = 4.11, *k* = 1050, *p* < 0.001). In addition, Y-HC compared to Y-BPD showed higher activation in a cluster that included the right caudate (peak voxel [x, y, z]: 22, 28, 0; *T* = 4.04, *k* = 100, *p* < 0.001) as well as in a cluster which comprised parts of the right middle and superior temporal gyri (peak voxel [x, y, z]: 42, −48, 10; *T* = 4.48, *k* = 430, *p* < 0.001). Similar to the latter finding, the group by age interaction revealed higher activation in the right middle and superior temporal gyri ((Y-HC>Y-BPD)>(A-HC>A-BPD), peak voxel [x, y, z]: 52, −46, 8; *T* = 3.69, *k* = 126, *p* < 0.001).

**Table 4:** Full-Factorial Analysis, whole brain results during other-directed aggression phase, *p* < 0.001 and cluster size *k* ≥ 88.

		***k***	***p***	***T***	***Z***	**MNI (peak voxel)**		
						***x***	***y***	***z***
Y-BPD > Y-HC	Left insula, left putamen, left rolandic operculum, left heschl gyrus, left inferior frontal gyrus opercular part, left middle temporal gyrus, left pallidum, left superior temporal gyrus, left precuneus, left calcarine fissure, left thalamus, left hippocampus	1,963	<0.001	5.03	4.95	−34	−48	8
Y-HC > Y-BPD	Right middle temporal gyrus, right superior temporal gyrus	430	<0.001	4.48	4.42	42	−48	10
	Right caudate	100	<0.001	4.04	4.00	22	28	0
(Y-BPD>Y-HC) > (A-BPD>A-HC)	Left insula, left putamen, left rolandic operculum, left inferior frontal gyrus opercular part, left inferior frontal gyrus triangular part, left pallidum	1,050	<0.001	4.11	4.06	−30	−12	8
(Y-HC>Y-BPD) > (A-HC>A-BPD)	Right middle temporal gyrus, right superior temporal gyrus	126	<0.001	3.69	3.66	52	−46	8

*Y-BPD, female adolescents with BPD; Y-HC, female adolescent healthy controls; A-BPD, female adults with BPD; A-HC, female adult healthy controls; k, cluster size; MNI, location of the peak voxel according to the Montreal Neurological Institute brain atlas*.

#### Self-directed aggression phase

When comparing Y-BPD to Y-HC, we found higher activation in a cluster including the left putamen and insula (peak voxel [x, y, z]: −32, −10, −2; *T* = 3.74, *k* = 178, *p* < 0.001) and, additionally, in the left middle temporal gyrus (peak voxel [x, y, z]: −50, −28, 0; *T* = 3.68, *k* = 93; *p* < 0.001). The reverse contrast (Y-HC>Y-BPD) as well as the group by age interactions did not reveal any significant effects (Table 5 and Figure 1C).

**Table 5:** Full-factorial analysis, whole brain results during self-directed aggression phase, *p* < 0.001 and cluster size *k* ≥ 88.

		***k***	***p***	***T***	***Z***	**MNI (peak voxel)**		
						***x***	***y***	***z***
Y-BPD > Y-HC	Left putamen, left insula	178	<.001	3.74	3.71	−32	−10	−2
	Left middle temporal gyrus	93	<0.001	3.68	3.65	−50	−28	0
Y-HC > Y-BPD	No significant results							
(Y-BPD>Y-HC) > (A-BPD>A-HC)	No significant results							
(Y-HC>Y-BPD) > (A-HC>A-BPD)	No significant results							

*Y-BPD, female adolescents with BPD; Y-HC, female adolescent healthy controls; A-BPD, female adults with BPD; A-HC, female adult healthy controls; k, cluster size; MNI, location of the peak voxel according to the Montreal Neurological Institute brain atlas*.

## Discussion

This is the first fMRI study investigating neural correlates of rejection-related feelings of anger and reactive aggression in adolescent BPD patients. Using a script-driven imagery setting to induce feelings of anger, and descriptions of subsequent aggressive reactions, we found increased activations in the left posterior insula and left dorsal striatum as well as in the inferior frontal gyrus and parts of the mentalizing network in female adolescents with BPD compared to female age-matched healthy controls. At least for the other-directed aggression phase, this pattern of activation could only be found in the adolescent sample suggesting specific alterations for adolescents with BPD. Together with previous studies, these findings suggest an enhanced emotional reactivity to interpersonal threat- or rejection-related situations early in the development of BPD. Since deficient emotion regulation has emerged an important mediator for aggression in BPD, the current findings support the need of early and specific interventions for affected adolescents.

Listening to anger-inducing descriptions of interpersonal rejection resulted in stronger activations in large clusters including the left posterior insula and the left dorsal striatum, mainly the putamen, in adolescents with BPD compared to healthy adolescents. Similar patterns of increased activations in the left insula and putamen, but also the middle temporal gyrus, were also found in adolescents with BPD during the descriptions of self-directed aggressive behaviors. Furthermore, the imagination of acting out aggressively against the rejecting person also caused elevated activations in large clusters including the left posterior insula and putamen, the middle temporal gyrus reaching into the superior temporal gyrus, the pallidum, precuneus, thalamus, hippocaumpus, and the inferior frontal gyrus. Notably the results of the group by age interaction indicate that the latter effect is characteristic for adolescents with BPD.

Increased left posterior insula activation indicates enhanced emotional reactivity to anger-inducing descriptions of interpersonal rejection and subsequent behavioral responses in terms of aggression directed against the own or the rejecting person in adolescents with BPD. The insula has been found to be crucially involved in the detection and processing of emotionally salient stimuli (Mühlberger et al., 2010). Therefore, it is not surprising that elevated insular activations are commonly reported in response to social exclusion or rejection in adults (Peyron et al., 2000; Eisenberger et al., 2003; Lieberman and Eisenberger, 2009) and adolescents (Masten et al., 2009). Since the posterior insula is part of the affective pain network, it's activations in the context of social rejection may be regarded as a neural correlate of social pain. Following this, the current findings suggest that interpersonal rejection leads to higher levels of social pain in adolescents with BPD than in healthy adolescents. In adult patients with BPD similarly elevated posterior insula activations in response to emotional stimuli have been interpreted as a neural correlate of deficient emotion regulation (Niedtfeld et al., 2010; Schulze et al., 2011). This is of interest since adolescents with BPD did not only show rejection-related increased, but also prolonged posterior insula responses that was also present during the description of subsequent other-directed and self-directed aggressive reactions suggesting deficits in emotion regulation capacities.

Support for the interpretation that interpersonal rejection may be more painful and hence salient for adolescents with BPD than healthy adolescents also comes from enhanced activations in the left dorsal striatum (putamen) as this region is involved in the coding of stimulus saliency (Zink et al., 2003). In addition, elevated activations in the thalamus and hippocampus in response to descriptions of other-directed aggressive reactions speak for enhanced elevated bottom up emotion generation (Reiman et al., 1997) and may indicate deficits in the processing of contextual information (Holland and Bouton, 1999; Liberzon and Abelson, 2016). Interestingly, stress effects on the hippocampus and the dorsal striatum are well documented and have been associated with alterations in related contextual and habitual memory processes (for review see de Quervain et al., 2017).

Contrary to our hypotheses and to previous studies in adults with BPD (Schulze et al., 2016), we did not find increased amygdala responses to the descriptions of interpersonal rejection, other-directed aggression, or self-directed aggression in female adolescents with BPD in the current study. As Herpertz et al. (2017) indeed found a heightened amygdala activation in male but not in female adults with BPD it remains unclear whether the lack of increased amygdala activation in the female adolescent sample in the present study is due to sex differences in amygdala responsivity to the current experimental manipulation or due to a lack in statistical power related to the rather small sample of adolescents (see below). It should also be taken into consideration that the lack of heightened amygdala activation in our paradigm might be specific to the adolescent sample. Previous structural MRI studies also could not find amygdala gray volume alterations (Chanen et al., 2008; Brunner et al., 2010) that have commonly been reported in samples of adult patients with BPD (see Schulze et al., 2016). Additionally, the fact that we found higher anger ratings not only in the adolescent patients, but also in the adolescent healthy controls may indicate a likewise increased emotional reaction—and amygdala activation—in the sample of the healthy controls as a reason for the lack of significant differences in this region between the two adolescent groups.

Interestingly, adolescents with BPD showed a heightened activation in the inferior frontal gyrus in response to descriptions of aggressive reactions against others. As the inferior frontal gyrus has been associated with different forms of self-control and self-regulation, including the regulation of emotion in general (Lieberman, 2007; Tabibnia et al., 2011) and of anger in particular (Fabiansson et al., 2012), this suggests strong efforts to control intense feelings of anger as well as aggressive impulses in adolescents with BPD. Furthermore, elevated activations in the middle temporal gyrus, superior temporal gyrus, and precuneus, regions that belong to the mentalizing network (Saxe and Powell, 2006), in adolescents with BPD could reflect a neural correlate of the tendency to hypermentalize or over-attribute emotions and intensions of others in adolescents with BPD (Sharp et al., 2011). Heightened activation in the precuneus might additionally reflect a stronger emotional reaction to interpersonal rejection in the adolescents with BPD in the present study, as a hyperactivation in the precuneus recently has been associated not only with mentalizing but also with the experience of social exclusion in adult healthy volunteers (Beyer et al., 2014) and with a heightened interpersonal rejection sensitivity in healthy adolescents (Masten et al., 2009).

On a behavioral level, the rating data did not reveal significant differences for the anger ratings between adolescents with BPD and healthy adolescents, which is contrary to our hypothesis. Interestingly healthy adolescents compared to adult healthy controls showed higher anger ratings when listening to descriptions of aggressive behavior, indicating a heightened emotional responding not only in adolescents with BPD but also in healthy adolescents. This might explain why the two adolescent groups did not differ in their anger ratings on the behavioral level, although they showed significant differences in neural activation.

Taken together, the fMRI data suggest a stronger salience of interpersonal rejection in adolescents with BPD associated with higher levels of social pain. Elevated activations in the thalamus and hippocampus suggest an even stronger activation of bottom-up emotion generation and memory retrieval despite high effort to mentalize and control feelings of anger. Considering heightened anger ratings also in healthy adolescents, the fMRI results indicate a level of emotion dysregulation in adolescents with BPD that goes beyond regular emotion regulation difficulties in adolescence (Guyer et al., 2016) suggesting deficits in the interplay of brain regions involved in the generation and regulation of negative emotions, which has been previously proposed as an important neural correlate of emotion dysregulation in BPD. Importantly, this is the first time that this has been shown in adolescents with BPD suggesting an early onset of a failure in emotion regulation.

The experimental paradigm and the possibility to compare the findings in adolescents to those of an adult sample are major advantages of the current study. Nevertheless, several shortcomings need to be mentioned. First, we were only able to include *N* = 20 female adolescents with BPD aged 15–17 years and future studies with larger samples of (medication-free) adolescents, a broader age spectrum and/or a longitudinal design are needed before strong conclusions can be drawn. Second, mainly for reasons of feasibility we only included female adolescents in the present study and thus were not able to investigate possible sex differences in neural correlates of anger and aggression in adolescents. As we recently reported distinct sex differences in the same task (Herpertz et al., 2017), further studies with male and female adolescents are needed.

Third, a clinical control group would be needed to address the specificity of the current findings for adolescents with BPD. Fourth, although the majority of our participants were medication-free, we cannot rule out that antidepressant medication in *N* = 4 adolescents with BPD may have affected amygdala activation (see Schulze et al., 2016 for negative effects of medication on amygdala activity in adult BPD patients). Fifthly a longitudinal investigation of the adolescent sample would be necessary to draw conclusions regarding the development of BPD symptomatology. Finally, we do not have information on feelings of loneliness, shame, guilt, or other negative emotions in response to our rejection-based anger induction and description of aggressive behaviors.

Our results suggest a stronger salience of interpersonal rejection and subsequent aggressive reactions in female adolescents with BPD compared to age-matched healthy female controls. A heightened emotional reactivity to interpersonal rejections might thus be already apparent at early developmental stages of BPD. A question that arises from the current findings is if the stronger emotional reaction to interpersonal rejection in adolescents with BPD is related to real experiences of former peer rejection. This aspect should be further addressed in future studies. In a therapeutic context it could be helpful for adolescents with BPD to develop functional strategies to regulate negative emotions, such as intense feelings of anger. So far, interventions from dialectical behavioral therapy for adolescents (DBT-A; Rathus and Miller, 2002), such as reality check or emotion regulation and stress tolerance skills, or mentalization-based interventions (MBT-A; Bateman and Fonagy, 2010; Rossouw and Fonagy, 2012) could be helpful to down-regulate high levels of emotional arousal already at a very early stage of BPD symptomatology. Learning adaptive emotion regulation strategies at an early stage of the disorder may reduce self-destructive and aggressive behaviors and thus increase the likelihood for positive interpersonal relationships and social functioning in general. Since interpersonal dysfunctions belong to the most persistent symptoms of BPD (Gunderson, 2007), specific and early treatments are of particular importance.

## Author contributions

MaK has contributed substantially to acquisition, analysis, and interpretation of the data. She has drafted the article. KU has contributed substantially to analysis and interpretation of the data. RB and MiK have contributed to conception and design as well as the interpretation of data. They have revised the manuscript critically for important intellectual content. SH has contributed substantially to acquisition of the data. She has revised the manuscript critically for important intellectual content. SCH and KB have contributed substantially to conception and design as well as the interpretation of data. They have revised the manuscript critically for important intellectual content. All authors gave final approval of the version to be published.

### Conflict of interest statement

The authors declare that the research was conducted in the absence of any commercial or financial relationships that could be construed as a potential conflict of interest.

## References

[B1] American Psychiatric Association (2013). Diagnostic and Statistical Manual of Mental Disorders, 5th Edn. Arlington, VA: American Psychiatric Publishing.

[B2] Barnow S. Stopsack M. Grabe H. J. Meinke C. Spitzer C. Kronmüller K.. (2009). Interpersonal evaluation bias in borderline personality disorder. Behav. Res. Ther. 47, 359–365. 10.1016/j.brat.2009.02.003 19278670

[B3] Bateman A. Fonagy P. (2010). Mentalization based treatment for borderline personality disorder. World Psychiatry 9, 11–15. 10.1002/j.2051-5545.2010.tb00255.x 20148147 PMC2816926

[B4] Bertsch K. Gamer M. Schmidt B. Schmidinger I. Walther S. Kästel T.. (2013). Oxytocin and reduction of social threat hypersensitivity in women with borderline personality disorder. Am. J. Psychiatry 170, 1169–1177. 10.1176/appi.ajp.2013.13020263 23982273

[B5] Bertsch K. Krauch M. Stopfer K. Haeussler K. Herpertz S. C. Gamer M. (2017). Interpersonal threat sensitivity in borderline personality disorder: an eye-tracking study. J. Pers. Disord. 31, 647–670. 10.1521/pedi_2017_31_273 28072041

[B6] Beyer F. Münte T. F. Krämer U. M. (2014). Increased neural reactivity to socio- emotional stimuli links social exclusion and aggression. Biol. Psychol. 96, 102–110. 10.1016/j.biopsycho.2013.12.008 24368143

[B7] Brunner R. Henze R. Parzer P. Kramer J. Feigl N. Lutz K.. (2010). Reduced prefrontal and orbitofrontal gray matter in female adolescents with borderline personality disorder: is it disorder specific?. Neuroimage 49, 114–120. 10.1016/j.neuroimage.2009.07.070 19660555

[B8] Buss A. H. Warren W. L. (2000). The Aggression Questionnaire Manual. Los Angeles, CA: Western Psychological Services.

[B9] Chanen A. M. Velakoulis D. Carison K. Gaunson K. Wood S. J. Yuen H. P.. (2008). Orbitofrontal, amygdala and hippocampal volumes in teenagers with first-presentation borderline personality disorder. Psychiatry Res. 163, 116–125. 10.1016/j.pscychresns.2007.08.007 18395424

[B10] de Quervain D. Schwabe L. Roozendaal B. (2017). Stress, glucocorticoids and memory: implications for treating fear-related disorders. Nature Rev. Neurosci. 18, 7–19. 10.1038/nrn.2016.155 27881856

[B11] Deffenbacher J. L. Oetting E. R. Thwaites G. A. Lynch R. S. Baker D. A. Stark R. S. (1996). State–trait anger theory and the utility of the trait anger scale. J. Couns. Psychol. 43:131 10.1037/0022-0167.43.2.131

[B12] Domsalla M. Koppe G. Niedtfeld I. Vollstädt-Klein S. Schmahl C. Bohus M.. (2013). Cerebral processing of social rejection in patients with borderline personality disorder. Soc. Cogn. Affect. Neurosci. 9, 1789–1797. 10.1093/scan/nst176 24273076 PMC4221221

[B13] Eisenberger N. I. Lieberman M. D. Williams K. D. (2003). Does rejection hurt? an fMRI study of social exclusion. Science 302, 290–292. 10.1126/science.1089134 14551436

[B14] Fabiansson E. C. Denson T. F. Moulds M. L. Grisham J. R. Schira M. M. (2012). Don't look back in anger: neural correlates of reappraisal, analytical rumination, and angry rumination during recall of an anger-inducing autobiographical memory. Neuroimage 59, 2974–2981. 10.1016/j.neuroimage.2011.09.078 22015853

[B15] Freyberger H. J. Spitzer C. Stieglitz R.-D. (1999). Fragebogen zu Dissoziativen Symptomen: FDS; ein Selbstbeurteilungsverfahren zur syndromalen Diagnostik dissoziativer Phänomene; deutsche Adaption der Dissociative Experience Scale (DES) von E. Bernstein-Carlsonu. FW, Putnam. Huber.

[B16] Goodman M. Hazlett E. A. Avedon J. B. Siever D. R. Chu K. W. New A. S. (2011). Anterior cingulate volume reduction in adolescents with borderline personality disorder and co-morbid major depression. J. Psychiatr. Res. 45, 803–807. 10.1016/j.jpsychires.2010.11.011 21145068

[B17] Gratz K. Roemer L. (2004). Multidimensional assessment of emotion regulation and dysregulation: development, factor structure, and initial validation of the difficulties in emotion regulation scale. J. Psychopathol. Behav. Assess. 26, 41–54. 10.1023/B:JOBA.0000007455.08539.94

[B18] Gunderson J. G. (2007). Disturbed relationships as a phenotype for borderline personality disorder. Am. J. Psychiatry 164, 1637–1640. 10.1176/appi.ajp.2007.07071125 17974925

[B19] Guyer A. E. Silk J. S. Nelson E. E. (2016). The neurobiology of the emotional adolescent: from the inside out. Neurosci. Biobehav. Rev. 70, 74–85. 10.1016/j.neubiorev.2016.07.037 27506384 PMC5074886

[B20] Harris J. A. (1997). A further evaluation of the aggression questionnaire: issues of validity and reliability. Behav. Res. Ther. 35, 1047–1053. 10.1016/S0005-7967(97)00064-8 9431736

[B21] Hayasaka S. Nicols T. E. (2004). Combining voxel intensity and cluster extent with permutation test framework. Neuroimage 23, 54–63. 10.1016/j.neuroimage.2004.04.035 15325352

[B22] Herpertz S. C. Nagy K. Ueltzhöffer K. Schmitt R. Mancke F. Schmahl C.. (2017). Brain mechanisms underlying reactive aggression in borderline personality disorder—sex matters. Biol. Psychiatry 82, 257–266. 10.1016/j.biopsych.2017.02.1175 28388995

[B23] Holland P. C. Bouton M. E. (1999). Hippocampus and context in classical conditioning. Curr. Opin. Neurobiol. 9, 195–202. 10.1016/S0959-4388(99)80027-0 10322181

[B24] Ibraheim M. Kalpakci A. Sharp C. (2017). The specificity of emotion dysregulation in adolescents with borderline personality disorder: comparison with psychiatric and healthy controls. Borderline Personal Disord. Emot. Dysregul. 4, 1. 10.1186/s40479-017-0052-x 28078089 PMC5223469

[B25] Kaess M. Brunner R. Chanen A. (2014). Borderline personality disorder in adolescence. Pediatrics 134, 782–793. 10.1542/peds.2013-3677 25246626

[B26] Koenigsberg H. W. Fan J. Ochsner K. N. Liu X. Guise K. G. Pizzarello S.. (2009). Neural correlates of the use of psychological distancing to regulate responses to negative social cues: a study of patients with borderline personality disorder. Biol. Psychiatry 66, 854–863. 10.1016/j.biopsych.2009.06.010 19651401 PMC2821188

[B27] Krause-Utz A. Elzinga B. M. Oei N. Y. Paret C. Niedtfeld I. Spinhoven P.. (2014). Amygdala and dorsal anterior cingulate connectivity during an emotional working memory task in borderline personality disorder patients with interpersonal trauma history. Front. Hum. Neurosci. 8:848. 10.3389/fnhum.2014.00848 25389397 PMC4211399

[B28] Lawrence K. A. Chanen A. M. Allen J. S. (2011). The effect of ostracism upon mood in youth with borderline personality disorder. J. Pers. Disord. 25, 702–714. 10.1521/pedi.2011.25.5.702 22023305

[B29] LeBoeuf A. Guilé J. Labelle R. Luck D. (2016). Neuroimagerie fonctionnelle chez l'adolescent avec un trouble de personnalité limite. Santé mentale au Québec 41, 141–162. 10.7202/1036969ar 27570955

[B30] Liberzon I. Abelson J. L. (2016). Context processing and the neurobiology of post-traumatic stress disorder. Neuron 92, 14–30. 10.1016/j.neuron.2016.09.039 27710783 PMC5113735

[B31] Lieberman M. D. (2007). Social cognitive neuroscience: a review of core processes. Annu. Rev. Psychol. 58, 259–289. 10.1146/annurev.psych.58.110405.085654 17002553

[B32] Lieberman M. D. Eisenberger N. I. (2009). Pains and pleasures of social life. Science 323, 890–891. 10.1126/science.1170008 19213907

[B33] Loranger A. W. (1999). International Personality Disorder Examination: IPDE; DSM-IV and ICD-10; Interviews: PAR. 8122958

[B34] Mancke F. Herpertz S. C. Bertsch K. (2015). Aggression in borderline personality disorder: a multidimensional model. Personal Disord. 6, 278. 10.1037/per0000098 26191822

[B35] Mancke F. Herpertz S. C. Kleindienst N. Bertsch K. (2017). Emotion Dysregulation and trait anger sequentially mediate the association between borderline personality disorder and aggression. J. Pers. Disord. 31, 256–272. 10.1521/pedi_2016_30_247 27064852

[B36] Masten C. L. Eisenberger N. I. Borofsky L. A. Pfeifer J. H. McNealy K. Mazziotta J. C.. (2009). Neural correlates of social exclusion during adolescence: understanding the distress of peer rejection. Soc. Cogn. Affect. Neurosci. 4, 143–157. 10.1093/scan/nsp007 19470528 PMC2686232

[B37] Mühlberger A. Wieser M. J. Gerdes A. B. Frey M. C. Weyers P. Pauli P. (2010). Stop looking angry and smile, please: start and stop of the very same facial expression differentially activate threat-and reward-related brain networks. Soc. Cogn. Affect. Neurosci. 6, 321–329. 10.1093/scan/nsq039 20460301 PMC3110429

[B38] Newhill C. E. Eack S. M. Mulvey E. P. (2009). Violent behavior in borderline personality. J. Pers. Disord. 23, 541–554. 10.1521/pedi.2009.23.6.541 20001173

[B39] Newhill C. E. Eack S. M. Mulvey E. P. (2012). A growth curve analysis of emotion dysregulation as a mediator for violence in individuals with and without borderline personality disorder. J. Pers. Disord. 26, 452–467. 10.1521/pedi.2012.26.3.452 22686232

[B40] Niedtfeld I. Schulze L. Kirsch P. Herpertz S. C. Bohus M. Schmahl C. (2010). Affect regulation and pain in borderline personality disorder: a possible link to the understanding of self-injury. Biol. Psychiatry 68, 383–391. 10.1016/j.biopsych.2010.04.015 20537612

[B41] Patton J. H. Stanford M. S. (1995). Factor structure of the Barratt impulsiveness scale. J. Clin. Psychol. 51, 768–774. 10.1002/1097-4679(199511)51:6<768::AID-JCLP2270510607>3.0.CO;2-1 8778124

[B42] Peyron R. Laurent B. Garcia-Larrea L. (2000). Functional imaging of brain responses to pain. a review and meta-analysis. Neurophysiol. Clin. 30, 263–288. 10.1016/S0987-7053(00)00227-6 11126640

[B43] Rathus J. H. Miller A. L. (2002). Dialectical behavior therapy adapted for suicidal adolescents. Suicide Life Threat Behav. 32, 146–157. 10.1521/suli.32.2.146.24399 12079031

[B44] Raven J. Raven J. C. Court J. H. (2003). Manual for Raven's Progressive Matrices and Vocabulary Scales. San Antonia, TX: Harcourt Assessment.

[B45] Reiman E. M. Lane R. D. Ahern G. L. Schwartz G. E. Davidson R. J. Friston K. J.. (1997). Neuroanatomical correlates of externally and internally generated human emotion. Am. J. Psychiatry 154, 918–925. 10.1176/ajp.154.7.918 9210741

[B46] Rossouw T. I. Fonagy P. (2012). Mentalization-based treatment for self-harm in adolescents: a randomized controlled trial. J. Am. Acad. Child Adolesc. Psychiatry 51, 1304–1313. 10.1016/j.jaac.2012.09.018 23200287

[B47] Santangelo P. S. Koenig J. Funke V. Parzer P. Resch F. Ebner-Priemer U. W.. (2017). Ecological momentary assessment of affective and interpersonal instability in adolescent non-suicidal self-injury. J. Abnorm. Child Psychol. 45, 1429–1438. 10.1007/s10802-016-0249-2 27995358

[B48] Saxe R. Powell L. J. (2006). It's the thought that counts: specific brain regions for one component of theory of mind. Psychol. Sci. 17, 692–699. 10.1111/j.1467-9280.2006.01768.x 16913952

[B49] Schmahl C. Herpertz S. C. Bertsch K. Ende G. Flor H. Kirsch P.. (2014). Mechanisms of disturbed emotion processing and social interaction in borderline personality disorder: state of knowledge and research agenda of the German Clinical Research Unit. Borderline Personal Disord. Emot. Dysregul. 1:12. 10.1186/2051-6673-1-12 26401296 PMC4579501

[B50] Schulze L. Domes G. Krüger A. Berger C. Fleischer M. Prehn K.. (2011). Neuronal correlates of cognitive reappraisal in borderline patients with affective instability. Biol. Psychiatry 69, 564–573. 10.1016/j.biopsych.2010.10.025 21195392

[B51] Schulze L. Schmahl C. Niedtfeld I. (2016). Neural correlates of disturbed emotion processing in borderline personality disorder: a multimodal meta-analysis. Biol. Psychiatry 79, 97–106. 10.1016/j.biopsych.2015.03.027 25935068

[B52] Scott L. N. Stepp S. D. Pilkonis P. A. (2014). Prospective associations between features of borderline personality disorder, emotion dysregulation, and aggression. Person. Disord. 5, 278. 10.1037/per0000070 24635753 PMC4099305

[B53] Serafini G. Pompili M. Borgwardt S. Houenou J. Geoffroy P. A. Jardri R.. (2014). Brain changes in early-onset bipolar and unipolar depressive disorders: a systematic review in children and adolescents. Eur. Child Adolesc. Psychiatry 23, 1023–1041. 10.1007/s00787-014-0614-z 25212880

[B54] Sharp C. Pane H. Ha C. Venta A. Patel A. B. Sturek J.. (2011). Theory of mind and emotion regulation difficulties in adolescents with borderline traits. J. Am. Acad. Child Adolesc. Psychiatry 50, 563–573. 10.1016/j.jaac.2011.01.017 21621140

[B55] Silbersweig D. Clarkin J. F. Goldstein M. Kernberg O. F. Tuescher O. Levy K. N.. (2007). Failure of frontolimbic inhibitory function in the context of negative emotion in borderline personality disorder. Am. J. Psychiatry 164, 1832–1841. 10.1176/appi.ajp.2007.06010126 18056238

[B56] Spielberger C. D. (1991). State-Trait Anger Expression Inventory: Staxi Professional Manual. Odessa: Psychological Assessment Resources.

[B57] Tabibnia G. Monterosso J. R. Baicy K. Aron A. R. Poldrack R. A. Chakrapani S.. (2011). Different forms of self-control share a neurocognitive substrate. J. Neurosci. 31, 4805–4810. 10.1523/JNEUROSCI.2859-10.2011 21451018 PMC3096483

[B58] Veague H. B. Hooley J. M. (2014). Enhanced sensitivity and response bias for male anger in women with borderline personality disorder. Psychiatry Res. 215, 687–693. 10.1016/j.psychres.2013.12.045 24485062

[B59] Whittle S. Chanen A. M. Fornito A. McGorry P. D. Pantelis C. Yücel M. (2009). Anterior cingulate volume in adolescents with first-presentation borderline personality disorder. Psychiatry Res. 172, 155–160. 10.1016/j.pscychresns.2008.12.004 19299113

[B60] Winsper C. Lereya S. T. Marwaha S. Thompson A. Eyden J. Singh S. P. (2016). The aetiological and psychopathological validity of borderline personality disorder in youth: a systematic review and meta-analysis. Clin. Psychol. Rev. 44, 13–24. 10.1016/j.cpr.2015.12.001 26709502

[B61] Wittchen H.-U. Zaudig M. Fydrich T. (1997). Strukturiertes Klinisches Interview für DSM-IV. Achse I und II. (Göttingen: Hogrefe).

[B62] Zanarini M. C. Frankenburg F. R. Reich D. B. Fitzmaurice G. Weinberg I. Gunderson J. G. (2008). The 10-year course of physically self-destructive acts reported by borderline patients and axis II comparison subjects. Acta Psychiatr. Scand. 117, 177–184 10.1111/j.1600-0447.2008.01155.x 18241308 PMC3884820

[B63] Zanarini M. C. Temes C. M. Ivey A. M. Cohn D. M. Conkey L. C. Frankenburg F. R.. (2017). The 10-year course of adult aggression toward others in patients with borderline personality disorder and axis II comparison subjects. Psychiatry Res. 252, 134–138. 10.1016/j.psychres.2017.02.054 28264784 PMC5438885

[B64] Zanarini M. C. Vujanovic A. A. Parachini E. A. Boulanger J. L. Frankenburg F. R. Hennen J. (2003). Zanarini Rating Scale for Borderline Personality Disorder (ZAN BPD): a continuous measure of DSM-IV borderline psychopathology. J. Pers. Disord. 17, 233–242. 10.1521/pedi.17.3.233.22147 12839102

[B65] Zink C. F. Pagnoni G. Martin M. E. Dhamala M. Berns G. S. (2003). Human striatal response to salient nonrewarding stimuli. J. Neurosci. 23, 8092–8097. 12954871 10.1523/JNEUROSCI.23-22-08092.2003 PMC6740503

